# Surgical reconstruction for spheno‐orbital meningioma extending into the sphenoid sinus with hyperostosis

**DOI:** 10.1002/ccr3.7705

**Published:** 2023-07-21

**Authors:** Toshihito Maki, Eiji Ito, Kiyoshi Saito, Ryuta Saito

**Affiliations:** ^1^ Department of Neurosurgery Kumiai Kosei Hospital Takayama Japan; ^2^ Department of Neurosurgery Nagoya University Graduate School of Medicine Nagoya Japan; ^3^ Department of Neurosurgery Fukushima Rosai Hospital Iwaki Japan

**Keywords:** hyperostosis, meningioma, skull base, sphenoid sinus, spheno‐orbital meningioma

## Abstract

Spheno‐orbital meningiomas (SOMs) are complex tumors that grow and extend into nearby structures. SOM tumor growth is often associated with hyperostosis caused by tumor cell infiltration and bone alteration. We describe the case of a 64‐year‐old man with SOM that extended into the sphenoid sinus without a direct connection between the intracranial and extracranial lesions. This report emphasizes the importance of identifying the growth patterns of SOMs and assessing the paranasal sinuses adjacent to the hyperostotic orbit walls from preoperative images.

## 1 INTRODUCTION

Spheno‐orbital meningiomas (SOMs) are rare tumors that display complex biological behavior and are distinct from other sphenoid wing tumors.^1^, ^2^, ^3^ SOMs are most commonly detected in the greater sphenoid wing with intraorbital extension into the temporal bone, temporal muscle, orbital apex, or cavernous sinus.^4^, ^5^ SOM tumor growth is often associated with hyperostosis, as tumor cells infiltrate and alter the bone.^1^, ^2^, ^3^, ^6^ While surgical techniques for SOMs have been established,^1^, ^2^, ^3^, ^7^ individually tailored surgical management is necessary to address the unique characteristics of each tumor. We present a case of SOM with hyperostosis that extended into the sphenoid sinus despite showing no direct connection to the intracranial and intraorbital lesions and required tailored surgical reconstruction for exophthalmos.

## 2 CASE PRESENTATION

**FIGURE 1:**
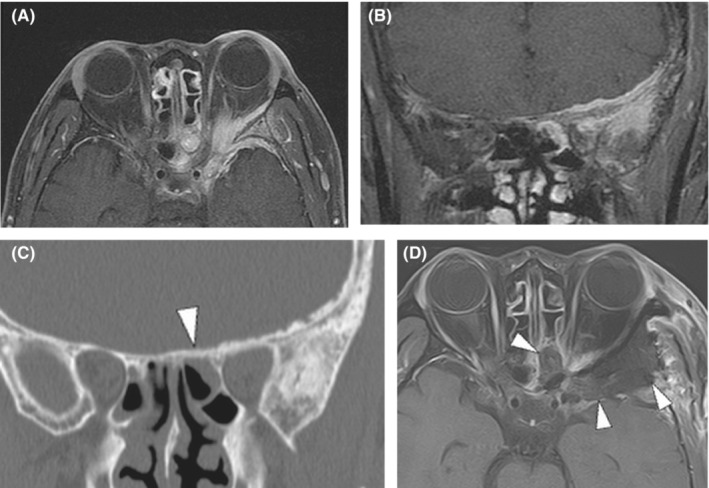
(A, B) Preoperative MRI demonstrating en plaque tumor along the greater wing of the left sphenoid bone and periorbita, extending into the temporal muscle and sphenoid sinus, leading to left exophthalmos. (C) Preoperative CT demonstrating a hyperostosis at the planum sphenoidale (arrowhead). (D) Postoperative MRI demonstrating symmetrical position of eyeball and galeal flap covering from left middle skull base to center anterior skull base (arrowheads). MRI, Magnetic resonance imaging; CI, Computed tomography.

A 64‐year‐old man presented with a 2‐year history of left‐sided proptosis. Magnetic resonance imaging (MRI) revealed an en plaque tumor along the greater wing of the left sphenoid bone and periorbita, extending into the temporal muscle. The lesion in the orbit compressed the orbital components and adjacent hyperostotic bones. Moreover, the MRI revealed a lesion in the sphenoid sinus separated from the intracranial and intraorbital lesions by thickened bone (Figure 1A,B). Computed tomography (CT) revealed remarkable hyperostosis of the left sphenoid bone, orbital walls, and planum sphenoidale (Figure 1C). Neuro‐ophthalmological examination showed no visual field defects or diplopia. Based on these findings, we diagnosed the lesion as a spheno‐orbital meningioma (SOM) causing exophthalmos. The tumor was surgically removed to confirm the histopathological features of the sphenoid sinus lesion and reconstruct the orbital walls.

**FIGURE 2:**
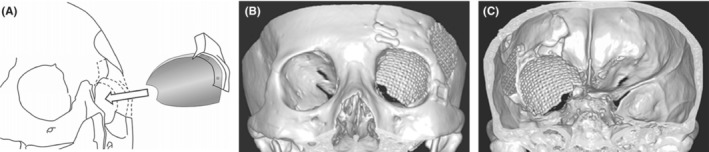
(A) Schematic illustration of orbital reconstruction using shaped titanium mesh fixed to the orbital rim. (B, C) Postoperative 3D‐CT demonstrating orbital reconstruction using remodeled titanium mesh. CT, Computed tomography.

A temporoparietal galeal flap was prepared for reconstruction under general anesthesia following a left coronal skin incision. Subsequently, a left frontotemporal craniotomy was performed. The intact orbital rim was removed, and the left anterior skull base and middle cranial base were epidurally exposed. After cutting the meningo‐orbital band, the bone around the optic canal and superior orbital fissure (SOF) was drilled away. The hyperostotic bone was removed to the maximum. The sphenoid sinus lesion was excised post the removal of the hyperostotic planum sphenoidale. No direct connection to the intracranial or intraorbital lesions was found. The intraorbital lesion was resected from the orbital compartment and the intracranial and temporal muscle lesions were thoroughly removed. After tumor resection, the dural defect was repaired using the femoral fascia lata. The lateral and superior walls of the orbit were reconstructed using titanium mesh fixed to the orbital rim (Figure 2). The bony defect in the anterior skull base was covered with the temporoparietal galeal flap to prevent cerebrospinal fluid (CSF) rhinorrhea (Figure 1D). Lumbar CSF drainage was not performed.

The patient's visual acuity deteriorated immediately after surgery but gradually improved. The patient was discharged without any neurological deficits or CSF leakage 1 week post the operation. No tumor recurrence was observed even 6 months after surgery.

Histopathological examination of the lesions in the sphenoid sinus and intracranial, intraorbital, and temporal muscles revealed a meningothelial meningioma with a MIB‐1 labeling index of 1%.

## 3 DISCUSSION

SOMs are a unique subgroup of meningiomas that exhibit a sheet‐like appearance and are associated with hyperostosis.^6^, ^8^, ^9^ When SOMs invade extracranial regions, they may exhibit two growth patterns: infiltration through natural openings, and hyperostosis formation. Infiltration through natural openings, such as the SOF, is a common route for tumor spread from the intracranial space to the orbit.^1^ On the other hand, hyperostosis formation occurs when meningiomatous cells invade the Haversian canals,^10^ leading to bone overgrowth and extracranial tumor infiltration.^9^ In the present case, the association of intraorbital and temporal muscle lesions with spheno‐orbital bone hyperostosis and planum sphenoidal hyperostosis in the sphenoid sinus lesion further emphasizes the importance of identifying these growth patterns in meningiomas. By assessing the paranasal sinuses adjacent to the hyperostotic orbital walls in preoperative images, clinicians can potentially identify signs of extracranial tumor invasion, aiding in accurate diagnosis and appropriate treatment planning.

The management of SOMs presents a challenge for clinicians because of the tumor's potential involvement of critical structures, such as the nerves in the SOF or intraorbital muscles, as well as the high recurrence rates associated with incomplete resection.^11^, ^12^, ^13^ Recent reports suggest that partial resection, in combination with postoperative radiation, may be a viable option in cases where radical resection is not feasible.^3^, ^6^, ^14^, ^15^ However, achieving aggressive resection of invasive tumors without causing neurological deficits remains a priority to reduce the likelihood of recurrence.^16^ Complete resection of SOMs can be difficult because of their wide extension in the skull base and involvement of the intraorbital compartment, further emphasizing the importance of a careful surgical approach.^17^ Published surgical techniques for SOMs^1^, ^2^, ^3^ highlight the importance of examination and removal of sphenoid sinus lesions for their management. Moving forward, the development of innovative surgical techniques and radiation therapy strategies may offer additional promise for improving the outcomes of patients with SOMs.

Proper management of skull base defects and dural closure after aggressive surgery for SOMs is crucial to prevent postoperative complications such as CSF leakage, which can be life‐threatening. However, achieving a watertight dural closure with a fascial patch graft can be challenging. Although free fat grafts may seem to be a convenient option, they have a low tolerance for infection. In this case, a vascularized temporoparietal galeal flap was used to cover the skull base defect because it is a well‐vascularized tissue that provides homogeneous thickness, making it a suitable option for skull base reconstruction. This technique is essential to eliminate dead space, ensure the sealing of the skull base, and prevent postoperative CSF leakage. The galeal flap adheres to the dural surface within several days, making indwelling lumbar spinal CSF drainage unnecessary.^18^ By utilizing appropriate surgical techniques such as the galeal flap, we can minimize the risk of postoperative complications and improve patient outcomes.

Orbital wall reconstruction after SOM surgery is crucial for restoring normal orbital volume and preventing ocular complications.^12^, ^19^, ^20^ Several materials, such as bone grafts,^3^, ^21^ porous polyethylene,^22^ titanium mesh,^23^, ^24^ and methylmethacrylate resin plates,^25^ have been used for orbital bony reconstruction. Among them, titanium mesh is considered the most suitable option, as it can be manually shaped to fit the orbital walls and be attached to the orbital rim. This produces good cosmetic and functional outcomes in the long term. Although other options, such as free bone graft, pedicled calvarial bone flap, and artificial bone, have been used, they each have limitations. Free bone grafts are prone to resorption over time, and shaping the fine orbital contour with pedicled calvarial bone can be difficult. It is important to note that although the use of hard tissue reconstruction for bony defects in the anterior cranial fossa has been a matter of controversy, orbital reconstruction after SOM surgery is necessary to prevent enophthalmos or pulsating exophthalmos. The use of a titanium mesh for orbital bony reconstruction is currently the most widely accepted technique for achieving optimal functional and cosmetic outcomes.^24^

The present case report has some limitations. Firstly, the case report represents a single case, limiting the findings' generalizability. Additionally, the lack of long‐term follow‐up data is a limitation, as it prevents us from providing more comprehensive information on the patient's outcomes and tumor recurrence. Lastly, a longer follow‐up period would have provided more robust information on long‐term outcomes and tumor recurrence rates.

In conclusion, preoperative assessment of the paranasal sinuses adjacent to hyperostotic orbital walls in SOMs may contribute to accurate diagnosis and appropriate treatment planning by identifying signs of extracranial tumor invasion. Additionally, tailored surgical management, including reconstruction techniques may achieve optimal outcomes for patients with SOMs.

## AUTHOR CONTRIBUTIONS

**Toshihito Maki:** Data curation; formal analysis; writing – original draft; writing – review and editing. **Eiji Ito:** Data curation; formal analysis; project administration; writing – original draft; writing – review and editing. **Kiyoshi Saito:** Conceptualization; data curation; supervision; writing – original draft; writing – review and editing. **Ryuta Saito:** Supervision; writing – original draft; writing – review and editing.

## FUNDING INFORMATION

This research received no specific grants from any funding agency in the public, commercial, or not‐for‐profit sectors.

## CONFLICT OF INTEREST STATEMENT

The authors received no financial support for this research and have no conflicts of interest to report regarding the materials and methods or findings of this paper.

## ETHICS STATEMENT

This study was performed in accordance with the ethical standards of the Japan Neurosurgical Society.

## CONSENT

Written informed consent was obtained from the patient for the publication of this case report and the accompanying data and images.

## Data Availability

Data sharing is not applicable to this article, as no datasets were generated or analyzed during the current study.
